# The Performance of DeepSeek R1 and Gemini 3 in Complex Medical Scenarios: Comparative Study

**DOI:** 10.2196/76822

**Published:** 2026-04-27

**Authors:** Maria Bajwa, Robert Hoyt, Dacre Knight, Maruf Haider

**Affiliations:** 1 MGH Institute of Health Professions, Boston, MA, United States; 2 Internal Medicine Department, Virginia Commonwealth University, 57 North 11th Street, Richmond, VA, 23298, United States, 1 8503845235; 3 Internal Medicine Department, University of Virginia, Charlottesville, VA, United States; 4 Internal Medicine Department, Carilion Roanoke Memorial Hospital, Roanoke, VA, United States

**Keywords:** large reasoning model, LRM, large language model, LLM, accuracy, medical scenario, DeepSeek R1, Gemini 3

## Abstract

**Background:**

Generative artificial intelligence models, especially reasoning large language models (LLMs), are gaining adoption in health care for diagnostic decision support and medical education. DeepSeek R1 is a reasoning LLM that generates extended chain-of-thought explanations to make its decision-making process more explicit. Traditional medical benchmarks often lack complexity and authenticity, motivating the adoption of scenario-rich datasets, such as the Massive Multitask Language Understanding Pro (MMLU-Pro) professional medicine subset, which provides multispecialty clinical vignettes for reasoning-centric evaluation.

**Objective:**

The objective of this study is to assess the diagnostic accuracy, reasoning quality, reasoning transparency, and practical usability of DeepSeek R1 and Gemini 3 Pro across closed- and open-ended clinical scenarios, with the intention of guiding their prospective application in practical clinical education and training. This evaluation was conducted by analyzing 162 diverse medical scenarios (both closed- and open-ended) from the MMLU-Pro health subset.

**Methods:**

In a 2-phase, dual-model evaluation, DeepSeek R1 and Gemini 3 Pro were applied to 162 matched clinical vignettes from the MMLU-Pro professional medicine subset spanning 21 specialties. Closed-ended, multiple-choice, and open-ended prompts were constructed for the same scenarios, and model outputs were coded for accuracy, reasoning steps, and citation behavior; descriptive statistics and the McNemar test were used to compare performance across formats.

**Results:**

DeepSeek R1 achieved an accuracy of 86.4% (140/162 scenarios) on closed-ended tasks and 80.9% (131/162) on open-ended questions across 162 clinical scenarios, indicating modest attenuation of performance when answer cues were removed. Gemini 3 Pro demonstrated 90.7% (147/162) closed-ended and 88.9% (144/162) open-ended accuracy on the same scenarios, showing a similar pattern of decreased performance without answer options. Error analysis indicated that incorrect answers typically involved longer reasoning chains, suggesting overthinking. In a structured review of open-ended responses, DeepSeek R1 produced an average of 18.7 (range 0‐52) references per case, with 5.2 unrelated references and 13.1 (range 3‐67) reasoning steps, whereas Gemini 3 Pro averaged 22.5 (range 12‐50) references, 1.9 (range 0‐8) unrelated references, and 4.4 (range 1‐10) reasoning steps per case.

**Conclusions:**

DeepSeek R1 demonstrated moderate-to-excellent accuracy and reasoning in evaluating both closed- and open-ended medical scenarios. In parallel, Gemini 3 Pro showed broadly comparable but distinct performance and reasoning patterns. While the closed-ended format may inflate accuracy due to cueing, the open-ended evaluation yielded richer insights into the fidelity of reasoning. Side-by-side evaluation of two large reasoning models highlights the importance of format, specialty, and citation behavior when considering clinical and educational use. Continued validation across a wider range of specialties and real-world contexts will enhance the model’s trustworthiness for diagnostic and teaching applications.

## Introduction

Generative artificial intelligence (AI) models, particularly large language models (LLMs), have demonstrated substantial advancements across various health care domains, including diagnostics, patient management, clinical documentation, and medical education [1,2]. With the emergence of reasoning-focused architectures, such as DeepSeek R1, the paradigm has shifted from text prediction to structured inference, characterized by chain-of-thought reasoning, mixture of experts, reinforcement learning, and more transparent decision paths [3-5]. Recent work has highlighted DeepSeek R1 as an open-source reasoning LLM with visualized decision pathways and low-cost deployment, and it has garnered growing interest in clinical decision support, patient engagement, and medical education; nevertheless, researchers have emphasized ongoing challenges related to hallucinations, modality limitations, and ethical integration into health care systems [6]. The features of reasoning LLMs are activated iteratively to answer a user’s zero-shot prompt, enabling clinicians and educators to use reasoning models in simple, instruction-based interactions [4,5,7]. In parallel, Gemini 3 Pro (Google Inc) has emerged as a state-of-the-art multimodal LLM that integrates strong language reasoning with image and structured-data understanding and has demonstrated high performance on broad academic benchmarks, including Massive Multitask Language Understanding Pro (MMLU-Pro), as well as medical examination–style question sets such as Medical Question Answering (MedQA), based on the United States Medical Licensing Examination (USMLE) [8]. Public benchmarks and technical reports describe Gemini 3 Pro (and related Med-Gemini variants) achieving competitive or superior accuracy to prior models across general knowledge and health care–oriented tasks, while also highlighting ongoing concerns about hallucinations, transparency, and responsible clinical deployment, similar to other frontier systems [8]. To our knowledge, no prior work has reported open-ended diagnostic performance and reasoning metrics for Gemini 3 Pro on the MMLU-Pro professional medicine benchmark subset.

Even with these changes, the use of LLMs in the real world still raises concerns about reliability, bias, replicability, and generalizability. Traditional evaluation benchmarks, most notably MedQA/USMLE multiple-choice questions (MCQs), have facilitated initial assessments of model performance, yet they are increasingly criticized for plateauing scores, susceptibility to cueing, testwiseness effects, and limited specialty representation. In these examination-style settings, models can sometimes infer correct answers from partial cues or test-taking strategies rather than demonstrating robust clinical reasoning, potentially overestimating their readiness for real-world use [9-12]. To address these limitations, MMLU-Pro, a modification of the original MMLU benchmark, was developed as a more robust and challenging multitask language dataset [13,14]. The MMLU-Pro professional medicine (health) subset provides scenario-based clinical vignettes across multiple specialties and is designed to increase diagnostic reasoning complexity, reduce test-taking artifacts, and broaden domain coverage compared with earlier examination-style benchmarks. A recent systematic review of 39 medical LLM benchmarks further quantified that examination-style “knowledge-based” benchmarks often report high accuracies (approximately 84%-90%), whereas more practice-based, clinically oriented benchmarks show substantially lower performance (approximately 45%-69%), particularly for clinical reasoning and safety, underscoring a persistent knowledge-and-practice performance gap and the need for richer, scenario-focused evaluation frameworks [15].

It is believed that the MMLU-Pro health subset increases the complexity of diagnostic reasoning, reduces test-taking artifacts, and provides a broader domain representation [13]. Previous research has evaluated the questioning capabilities of LLMs [16]. Much of this work has relied on single-format multiple-choice or short-answer medical question-answering benchmarks, emphasizing aggregate accuracy on examination-style items rather than detailed analyses of clinical reasoning processes [16-18]. Nonetheless, limited research has used MMLU-Pro in a scenario-rich, dual-format assessment that encompasses both constrained (closed-ended) and expressive (open-ended) reasoning tasks. In this context, closed-ended tasks require the model to select an answer from predefined options (for example, choosing a single best diagnosis from 4 MCQ choices), whereas open-ended tasks require the model to generate a free-text diagnosis and supporting reasoning without explicit answer cues, more closely approximating how clinicians articulate and justify diagnostic judgments in practice. [15-18,] The MMLU-Pro dataset is unique in that it offers 10 potential answers for a model to choose from. Based on prior work, we hypothesized that the question format of open- and closed-ended questions would have a meaningful impact on model performance in complex medical scenarios and clinical reasoning [17,18].

We align our study with the Transparent Reporting of a Multivariable Model for Individual Prognosis or Diagnosis—Large Language Model (TRIPOD-LLM) guidelines to offer new insights into model behavior across diverse clinical tasks [19]. TRIPOD-LLM guidance ensured a structured protocol to highlight transparency, reproducibility, and standardized reporting across all evaluation stages [19]. The intended audience for this work includes clinicians, educators, and AI researchers interested in using or evaluating LLMs or large reasoning models (LRMs) for diagnostic decision support, curriculum design, and medical reasoning education. A completed TRIPOD-LLM compliance checklist, mapping each reporting item to corresponding manuscript sections, is provided in Multimedia Appendix 1.

The objective of this study is to assess the diagnostic accuracy and reasoning quality of DeepSeek R1 and Gemini 3 Pro with the intention of guiding their prospective application in practical clinical education and training. Gemini 3 was selected as a comparable LLM. More details on this model are provided in the Methods section. We aimed to (1) compare model accuracy and behavior across prompt formats using structured evaluation criteria, (2) assess variability in performance across clinical domains, and (3) characterize types of reasoning and interpretability errors to identify limitations in clinical reasoning and generalizability for both models.

## Methods

**Figure 1.**
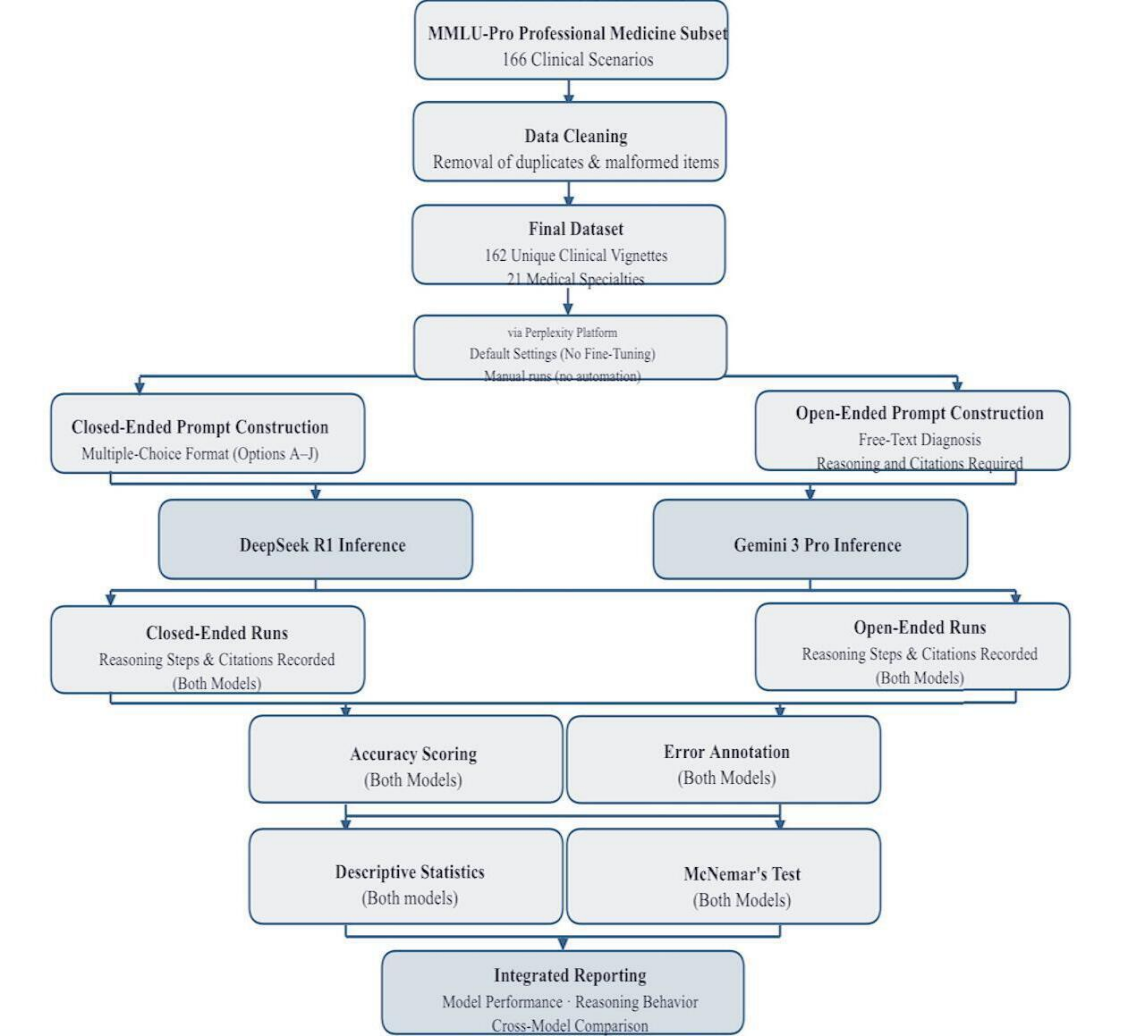
Illustration of the study design, with 2 large language models.

We conducted a 2-phase, protocol-driven, 2-model exploratory evaluation of DeepSeek R1 and Gemini 3 Pro to test their performance and diagnostic behaviors across structured clinical vignettes [14]. (Figure 1)

### Data Source and Preparation

The MMLU-Pro dataset contains over 12,000 questions in 14 categories [13]. We identified the subcategory of “professional medicine,” comprising 166 complex medical scenarios categorized across 21 specialties and spanning a spectrum of real-world clinical contexts, from primary care to subspecialty practice. Duplicates and erroneously formatted cases were removed. Duplicates were defined as items with identical clinical stems and answer keys and were collapsed to a single representative scenario. Erroneously formatted cases were those with obvious structural problems (eg, missing answer options, incomplete vignette text, or clearly mismatched options in the MMLU-Pro files) and were excluded. A final set of 162 scenarios served as the basis for both study phases, each presented as a clinical vignette to assess diagnostic reasoning. We regarded the provided key to the answers as the definitive source of information. For closed-ended items, responses were scored as correct if the selected letter matched the keyed answer. For open-ended items, free-text diagnoses were considered correct if they exactly matched or were clear clinical synonyms of the keyed diagnosis (eg, “acute myocardial infarction” vs “myocardial infarction”), as determined by author review. Three practicing clinicians on the author team independently resolved any ambiguous cases by consensus discussion. No imputation was performed; error breakdowns are reported for all scenarios, and the dataset was last modified in June 2024. An example scenario is shown in Textbox 1.

Textbox 1. **Example scenario (#6037).**

A 47-year-old man is brought to the emergency department 2 hours after the sudden onset of shortness of breath, severe chest pain, and sweating. He has no history of similar symptoms. He has hypertension treated with hydrochlorothiazide. He has smoked one pack of cigarettes daily for 30 years. His pulse is 110/min, respirations are 24/min, and blood pressure is 110/50 mm Hg. A grade 3/6 diastolic blowing murmur is heard over the left sternal border and radiates to the right sternal border. Femoral pulses are decreased bilaterally. An ECG shows left ventricular hypertrophy. Which of the following is the most likely diagnosis? ['A. Acute myocardial infarction' B. 'Congestive heart failure' C. 'Angina pectoris' D. 'Aortic dissection' E. 'Mitral valve prolapse' F. 'Esophageal rupture' G. 'Hypertensive crisis' H. 'Thoracic aortic aneurysm' I. 'Pulmonary embolism' J. 'Aortic stenosis']

### Models Evaluated

The evaluation was conducted on DeepSeek R1 (January 2025 release), a publicly available, open-source LRM [20]. We used an uncensored version of DeepSeek hosted by a well-known AI search engine platform, Perplexity AI (Perplexity AI, Inc) [21,22]. No model fine-tuning, postprocessing, or temperature modification was applied. No post hoc calibration, bias correction, or output pruning was performed.

We also evaluated Gemini 3 Pro, a proprietary large multimodal model with advanced reasoning capabilities (version 1, released November 18, 2025) [23]. The Gemini 3 series excels at complex tasks involving text, images, video, audio, and code. Technical features include a 1-million-token context window and native multimodal support. The models support up to 3000 images or 45 minutes of video in a single prompt. The knowledge cutoff date is January 2025 [23]. Gemini 3 Pro was accessed through the same conversational interface using identical prompts, scenarios, and default settings, with no fine-tuning, temperature modification, or post hoc calibration. Both models were run manually (no automation), one scenario per prompt, for closed- and open-ended questions.

### Computational Resources

Both DeepSeek R1 and Gemini 3 Pro were accessed via Perplexity AI’s web chat platform (model version not explicitly labeled; presumed latest release at the time of evaluation). All runs used the same conversational interface, prompts, and default model settings, with no fine-tuning, temperature, or max-token modification, post hoc calibration, bias correction, or output pruning. Inference was executed on Perplexity’s standard cloud infrastructure, with typical per-query latency of approximately 4 to 20 seconds; detailed hardware configuration, server location, and floating-point throughput are unavailable. DeepSeek R1 was evaluated from March 6 to 10, 2025, for closed-ended questions, and from March 12 to 15, 2025, for open-ended questions, using default settings (temperature 1.0, max tokens 2048) and one scenario per prompt, as batch execution was not available. Gemini 3 Pro was evaluated on the same 162 scenarios under identical manual execution procedures, from January 17 to 22, 2026, for closed-ended questions and February 12 to 25, 2026, for open-ended questions, again with one scenario per prompt and no automation agent.

### Evaluation Protocol

The evaluation protocol closely follows TRIPOD-LLM’s recommendations for rigorous, open LLM evaluation in health contexts and is reported accordingly [19]. The study evaluated the performance of DeepSeek R1 and Gemini 3 on the MMLU-Pro professional medicine subset across 2 phases, using both closed-ended MCQs and open-ended questions [20-23]. To interact with the LRM, prompts were designed with clear, reproducible instructions; in the open-ended format, the only modification was removing answer choices and phrasing cues. The full 2-phase protocol was then repeated with Gemini 3 Pro using the same prompts, scenarios, and execution procedures.

In both phases and both LRMs, disagreements with the MMLU-Pro key were recorded and reported without further resolution for future review. All queries were run manually without the aid of an automation agent. A structured error-annotation system, described in the Qualitative Analysis section, was applied to all incorrect answers to characterize model behavior. Relatedness, nonalignment to the answer keys, or hallucination of citations were coded by the principal investigator and reviewed for consensus among the author team.

### Closed-Ended Phase

The model’s chosen answer (A-J) and generated rationale and literature citations were recorded for each scenario. We measured the actual reasoning steps for closed- and open-ended questions. Model answers were compared to the official MMLU-Pro answer key. For each incorrect response, an error category was then assigned using the structured taxonomy described below, and the associated citation count and reasoning-step count were recorded for subsequent descriptive analysis.

The closed-ended prompt was as follows: “As a medical consultant, you will respond to questions about various medical scenarios. This is a multiple-choice question with up to 10 options. Select the answer that is most likely. Report the correct answer A-J. Then, provide your reasoning steps and cite relevant literature.” The input structure had (1) the prompt text, (2) a full scenario vignette, and (3) a list of answer choices (A, B,… J).

### Open-Ended Phase

In the second phase, scenarios were reformatted as open-ended prompts that required diagnosis and differential diagnosis without provided options. For each item, the original MMLU-Pro vignette stem was preserved verbatim, while the last clause, “Which of the following is the most likely diagnosis?” (or equivalent) and the accompanying answer list were removed so that the model received the same clinical information in both phases but without explicit options in the open-ended condition. Both DeepSeek R1 and Gemini 3 Pro were prompted with the open-ended version using the following prompt: “As a senior clinician, you have received requests for consultation on various medical scenarios. Provide a diagnosis and differential diagnosis for each case. Then, explain your reasoning and cite relevant literature.” The input structure included (1) the above prompt text, (2) a full scenario vignette, and (3) no answer options.

The output included diagnosis, differentials, rationale, citations, and possible hallucinations and was recorded. Outputs were assessed by 2 board-certified clinician authors (MH and DK), each reviewing half the cases for accuracy and alignment with the ground truth, and discrepancies were resolved by a third clinician author (RH). Afterwards, the same structured error-annotation framework used in the closed-ended phase was used to assign the errors in this phase. Each incorrect open-ended response was assigned to a single error category, and its citation count and reasoning-step count were extracted using the same procedures as for closed-ended items.

### Statistical Analysis

Descriptive statistics, including frequency and proportion, were calculated for model accuracy, citation count, number of reasoning steps, error frequencies, and specialty-specific performance. For both formats, each scenario was scored as correct (1) or incorrect (0) based on a comparison with the MMLU-Pro answer key, as described above, and overall accuracy was calculated as the proportion of correctly answered items out of 162. Citation counts were obtained by manually counting the distinct reference links or source objects produced by the models in each response. The number of reasoning steps was taken from the model’s own structured reasoning output (eg, “Step 1… Step 2…”), using the step count reported in the response when available and, when absent, by counting discrete reasoning statements in the model’s explanation. Each vignette was mapped to a single specialty using the professional medicine labels in MMLU-Pro, supplemented by manual assignments when necessary, to derive specialty-specific accuracy. Each scenario was scored as correct or incorrect, and model accuracy was compared between closed- and open-ended formats. The McNemar test was then applied to the paired binary data of closed- vs open-ended questions for each model to determine whether there was a statistically significant difference in results between the 2 related groups, as recommended in the statistical literature for within-subject categorical comparisons [24,25]. No formal statistical comparisons were conducted between formats for citation or reasoning metrics due to differences in sampling completeness and the limited number of questions. All statistical tests were 2-tailed, and results were considered significant at *P*<.05. No power calculation was performed due to the fixed number of scenarios (n=162).

### Qualitative Analysis

To categorize the errors, we developed a structured taxonomy for the error annotation system by analyzing incorrect outputs thematically. All queries were executed manually without the use of automated agents. The error annotation framework was informed by prior literature [26] and refined through consensus among the research team. Initial error categories were drafted based on observed response patterns and iteratively revised to ensure consistent interpretation. All incorrect outputs were reviewed using the original dataset, including reasoning steps, citation counts, and specialty labels, and disagreements were resolved through team discussion.

### Ethical Considerations

No patient or human subject data were used. Therefore, approval from the ethical review board was not required. Model, prompt, and grading protocols are available upon request.

## Results

### Model Performance

DeepSeek R1 and Gemini 3 Pro were each evaluated on 162 clinical scenarios from the MMLU-Pro professional medicine subset spanning multiple medical specialties. Both models were assessed on the same cases using closed-ended (multiple-choice) and open-ended (free-response) formats. The largest specialty groups included primary care, emergency medicine, pediatrics, obstetrics and gynecology, and neurology, each contributing 10 or more cases. Model responses were classified as correct or incorrect to assess performance across question formats and clinical domains. Specialty-level distribution and diagnostic accuracy for specialties with 6 or more scenarios are summarized in Table 1; the full set of specialties is provided in Multimedia Appendix 2.

**Table 1.** Diagnostic accuracy by clinical specialty for DeepSeek R1 and Gemini 3 Pro across closed- and open-ended formats for specialties with 6 cases or more in the question set. The last row provides an average per column for the 8 specialties.

Clinical specialty	DeepSeek R1 (n=162)		Gemini 3 Pro (n=162)	
	Closed-ended correct, n (%)	Open-ended correct, n (%)	Closed-ended correct, n (%)	Open-ended correct, n (%)
Primary care (n=33)	28 (84.8)	26 (78.8)	33 (100.0)	32 (97.0)
Emergency medicine (n=26)	25 (96.2)	22 (84.6)	22 (84.6)	23 (88.5)
Pediatrics (n=17)	16 (94.1)	16 (94.1)	15 (88.2)	15 (88.2)
Obstetrics and gynecology (n=11)	8 (72.7)	7 (63.6)	9 (81.8)	9 (81.8)
Neurology (n=10)	9 (90.0)	10 (100.0)	10 (100.0)	9 (90.0)
Statistics (n=9)	7 (77.8)	8 (88.9)	9 (100.0)	7 (77.8)
Infectious disease (n=7)	6 (85.7)	4 (57.1)	6 (85.7)	7 (100.0)
Endocrinology (n=6)	6 (100.0)	6 (100.0)	6 (100.0)	6 (100.0)
Average score for 8 specialties, %	87.8	83.5	92.6	90.5

**Figure 2.**
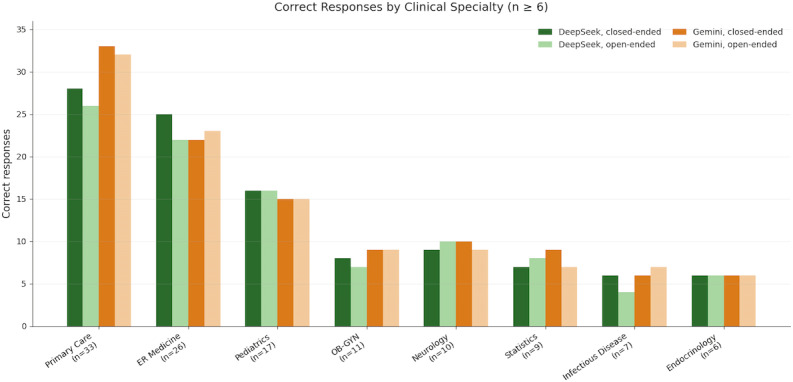
Diagnostic accuracy of DeepSeek R1 and Gemini 3 Pro across clinical specialties with ≥6 evaluated scenarios.

DeepSeek R1 answered 140 of 162 scenarios correctly in the closed-ended format (86.4%), whereas Gemini 3 Pro answered 147 (90.7%). Accuracy was generally high across clinical domains, with moderate variation by specialty (Table 1). When answer options were removed, performance declined for both models: DeepSeek R1 answered 131 scenarios correctly (80.9%), and Gemini 3 Pro answered 144 (88.9%). The reduction in accuracy associated with removing answer options was greater for DeepSeek R1 than for Gemini 3 Pro (5.5 vs 1.8 percentage points). Accuracy varied across specialties, with obstetrics and gynecology demonstrating the lowest average accuracy (75.2%) (Table 1; Figure 2).

### Paired Accuracy Comparison

To assess whether diagnostic accuracy differed between question formats, the McNemar test was applied to paired responses for each model across the 162 scenarios. For DeepSeek R1, the difference between closed-ended and open-ended accuracy was not statistically significant (*χ*²_1_=3.37; *P*=.07). Similarly, Gemini 3 Pro showed no significant difference between formats (*χ*²_1_=0.21; *P*=.65). These findings indicate that removing answer options did not produce a statistically significant change in diagnostic accuracy for either model.

### References and Reasoning Steps

Table 2 demonstrates that, in the open-ended condition, DeepSeek R1 included more references and reasoning steps than Gemini 3 Pro but had lower accuracy. One response (Q#6111) represented an extreme outlier, containing 70 unrelated references, largely focused on HIV literature rather than the statistical reasoning required by the scenario. Citation and reasoning metrics are reported for open-ended responses only (n=162 per model); closed-ended citation and reasoning data were not collected comprehensively for both models. Because our primary interest was in understanding diagnostic reasoning behavior in unconstrained, real-world–like settings, we prioritized coding of citations and reasoning steps for open-ended responses, whereas closed-ended outputs were evaluated primarily for accuracy and error patterns rather than detailed citation analysis.

**Table 2.** References, unrelated references, and reasoning steps per model on open-ended questions.

	References		Unrelated references	Steps	
Model	Mean (range)	Median	Unrelated references, mean (range)	Mean (range)	Median
DeepSeek	33.1 (0‐84)	30	3.1 (0‐70)	10.7 (3‐34)	10
Gemini	22.5 (12‐50)	15	1.9 (0‐8)	4.4 (1‐10)	4

### Error Analysis

Errors were unevenly distributed across specialties and question formats. For DeepSeek R1, closed-ended errors were concentrated in primary care (n=5; questions #6249, #6432, #6493, #6501, #6507), obstetrics and gynecology (n=3; questions #6183, #6307, #6664), and general surgery (n=3; questions #6491, #6503, #6611). In the open-ended condition, errors increased in several of these specialties and also appeared in emergency medicine (n=4; questions #6310, #6544, #6748, #6793), infectious disease (n=3; questions #6236, #6242, #6682), and psychiatry (n=2; questions #6497, #6674), specialties that had few or no errors in the closed-ended format. Overall, DeepSeek R1 produced more errors in the open-ended format (n=31) than in the closed-ended format (n=22), with 8 specialties showing reduced accuracy when answer options were removed.

For Gemini 3 Pro, the distribution differed. Closed-ended errors occurred in emergency medicine (n=3; questions #6310, #6557, #6796), pediatrics (n=2; questions #6252, #6430), obstetrics and gynecology (n=2; questions #6183, #6307), general surgery (n=2; questions #6503, #6608), and psychiatry (n=2; questions #6112, #6674). In contrast to DeepSeek R1, the open-ended format improved performance in several of these areas: 8 questions answered incorrectly in the closed-ended condition were answered correctly in the open-ended condition. New open-ended errors appeared primarily in specialties that had no closed-ended failures, including oncology (n=2; questions #6187, #6618), statistics (n=2; questions #6111, #6664), and trauma surgery (n=1; question #6043). Three specialties—endocrinology, nephrology, and immunology—showed no errors across either model or format.

Persistent errors further clarified differences between models. DeepSeek R1 answered 17 items incorrectly in both formats (questions #6045, #6183, #6184, #6249, #6252, #6307, #6310, #6432, #6491, #6493, #6501, #6503, #6550, #6611, #6664, #6672, #6682), spanning primary care, obstetrics and gynecology, general surgery, and infectious disease. These persistent errors represented 77% (17/22) of DeepSeek R1’s closed-ended failures and 55% (17/31) of its open-ended failures, suggesting that many of its mistakes reflected format-independent uncertainty rather than sensitivity to question type.

Gemini 3 Pro demonstrated fewer persistent errors (n=7; questions #6252, #6307, #6503, #6550, #6557, #6674, #6796), concentrated mainly in emergency medicine, obstetrics and gynecology, and psychiatry. These accounted for 47% of its closed-ended failures, indicating that Gemini’s performance was more responsive to question type. Four questions (#6252 [pediatrics], #6307 [obstetrics and gynecology], #6503 [general surgery], and #6550 [vascular surgery]) were answered incorrectly by both models across both formats, suggesting question-level complexity or ambiguity rather than model-specific limitations (Table 3).

**Table 3.** Error taxonomy with representative examples for DeepSeek R1 and Gemini 3 Pro. Error taxonomy was applied to all incorrect open-ended responses for both models and to incorrect closed-ended responses for DeepSeek R1. “Not observed” indicates that the error type was not identified in any response for that model.

Error type	Description	DeepSeek R1 example	Gemini 3 Pro example
E1: Reasonable but nonoption answer	Clinically valid response that diverges from the benchmark key	Question #6118 (primary care): model provided a plausible but unlisted management response in the open-ended format; correct in the closed-ended condition (both closed and open formats).	Question #6043 (trauma surgery): model selected a clinically defensible approach not aligned with the MMLU-Pro answer key (both closed and open formats).
E2: Overthought/ contradictory reasoning	Excessively long or internally inconsistent reasoning chain	Question #6491 (general surgery): closed-ended response generated 56 reasoning steps before reaching an incorrect conclusion, with internal contradictions across steps (both closed and open formats).	Not observed.
E3: Citation hallucination	Fabricated or unverifiable citations presented as real references	Not observed.	Not observed.
E4: Missing differentials	Failure to address relevant diagnostic alternatives	Question #6672 (allergy immunology): open-ended response did not consider AM cortisol measurement as a diagnostic step, which was central to the correct answer (both closed and open formats).	Question #6104 (neurology): open-ended response failed to incorporate key neurological differentials, leading to an incomplete diagnostic approach (both closed and open formats).
E6: Circular logic	Repetition of reasoning without progress toward diagnosis	Question #6310 (emergency medicine): closed-ended response generated 31 reasoning steps that restated the clinical scenario repeatedly without narrowing the differential or reaching a defensible conclusion (both closed and open formats).	Not observed.
E7: Ambiguous/overloaded output	Diffuse reasoning with excessive or tangential information	Question #6254 (genetics): closed-ended response produced 46 citations for a genetics question, with tangential references diluting the clinical focus of the answer (both closed and open formats).	Not observed.

In several cases across both models, responses classified as incorrect were judged by the authors to represent clinically reasonable alternatives, reflecting updated guidelines or equivalent management strategies not captured by the MMLU-Pro answer key. This pattern was particularly prominent for Gemini 3 Pro, where 17 of 18 open-ended errors (94%) were classified as E1 (reasonable but nonoption answer). These findings suggest that benchmark accuracy alone may not fully capture clinically valid reasoning in LLMs.

### Qualitative Analysis of the Errors

To understand the mechanisms underlying incorrect responses, we applied the structured error taxonomy described in the Methods section to all incorrect open-ended responses from both models and to closed-ended incorrect responses from DeepSeek R1. Each error was categorized into one of six predefined types: reasonable but nonoption answer (E1), overthought or contradictory reasoning (E2), citation hallucination (E3), missing differentials (E4), circular logic (E6), and ambiguous or overloaded output (E7) (Table 2).

Across the dataset, the most common error type was E1 (reasonable but nonoption answer), followed by E2 (overthought or contradictory reasoning) and E4 (missing differentials). Citation hallucination (E3) was not observed in either model, indicating that the models’ references were generally grounded in real sources. Overthought reasoning and ambiguous outputs (E2 and E7) were characterized by unusually long or diffuse reasoning chains. In contrast, E4 (missing differentials) occurred exclusively in open-ended responses for both models, consistent with the unconstrained prompt structure, allowing broader diagnostic elaboration without predefined answer options.

The two models exhibited distinct error profiles. DeepSeek R1 demonstrated a broader range of error types, including E1, E2, E4, E6, and E7. Several of these errors reflected instability in reasoning, such as excessively long chains of reasoning (E2) or circular diagnostic logic (E6). In contrast, Gemini 3 Pro’s errors were overwhelmingly classified as E1, with 17 of 18 open-ended errors representing clinically plausible answers that diverged from the benchmark key. Only a single Gemini response showed a missing-differential error (E4). These contrasting patterns suggest that the models differed not only in overall accuracy but also in their failure modes: DeepSeek R1’s errors more often reflected reasoning instability under uncertainty, whereas Gemini 3 Pro more frequently produced clinically plausible answers that diverged from the benchmark answer key (Table 3).

## Discussion

### Principal Findings

Our evaluation demonstrated that Gemini 3 Pro achieved higher overall performance (90.7% closed-ended; 88.9% open-ended) than DeepSeek R1 (86.4% closed-ended; 80.9% open-ended). Accuracy declined modestly when answer options were removed for both models; however, paired analysis showed that this difference was not statistically significant.

DeepSeek R1 produced longer reasoning chains, but these did not improve accuracy. Its errors were distributed across multiple reasoning categories, including overthought reasoning, circular logic, and missing differentials. In contrast, most Gemini 3 Pro errors, particularly in open-ended responses, were classified as clinically reasonable answers. Consistent with prior evaluations of medical language models, a substantial proportion of incorrect responses in this study represented clinically plausible alternatives rather than clearly incorrect reasoning [9,12].

Specialty-level analysis demonstrated moderate variability in model performance across domains. Both models performed consistently well in pediatrics, neurology, and endocrinology, whereas lower accuracy was observed in specialties such as obstetrics and gynecology and primary care. Persistent errors across both question formats were relatively limited but suggest that certain scenarios posed intrinsic reasoning challenges independent of question type. The number of questions per specialty was small, limiting interpretation and generalizability. Accuracy reported here was consistent with, or exceeded, previous reports on prominent LLMs on medical benchmarks, including DeepSeek R1 and Gemini, as well as GPT-4, MedPaLM, and Claude [26-29].

Our results also contribute to a growing body of research examining how chain-of-thought reasoning affects model performance. While reasoning transparency is often considered a strength of LRMs, recent studies suggest that longer reasoning chains do not necessarily improve accuracy [30,31]. The reasoning patterns observed in DeepSeek R1 responses support this observation.

These results have implications for educational integration, a prominent opportunity for LRMs. When used with instructor oversight, reasoning models may support case-based learning, differential diagnosis exercises, and discussions of diagnostic reasoning processes. This transparency enables clinicians and educators to review diagnostic logic, reasoning chains, differentials, and literature-supported explanations as practical case-based learning resources [32,33].

Benchmark accuracy alone provides an incomplete assessment of a model’s capabilities. For example, Gemini 3 Pro achieved higher accuracy with shorter reasoning outputs, suggesting that concise reasoning structures may sometimes reflect more stable inference processes. From a clinical safety perspective, the results reinforce current guidance that LLMs should not be used as independent diagnostic decision systems. Even highly accurate models occasionally produce reasoning errors or plausible but incorrect conclusions. Current expert recommendations emphasize that AI-generated clinical reasoning should be interpreted as decision-support information rather than authoritative clinical guidance and should always be reviewed by qualified clinicians.

### Strengths and Limitations

This study has several strengths. First, it evaluated 2 LRMs using the same dataset, prompts, and evaluation protocol, allowing direct comparison of model behavior across identical clinical scenarios. Second, the dual-format design, incorporating both closed-ended and open-ended prompts, enabled assessment of diagnostic reasoning with and without answer cueing. This technique provides a more realistic evaluation of free-text clinical reasoning. The analysis integrated quantitative performance metrics with a structured qualitative error taxonomy, facilitating a more detailed analysis.

This study also has several limitations. First, the evaluation was limited to the professional medicine subset of the MMLU-Pro dataset. Although this benchmark includes complex clinical scenarios across multiple specialties, it does not fully capture the longitudinal context or multimodal information present in real-world clinical practice. The number of available questions limited the statistical power for specialty-level analyses. Qualitative error analysis only focused on a targeted subset of model outputs. Open-ended answers for one model were only reviewed by one physician reviewer. Ideally, they should be reviewed by two reviewers, with a third involved if there is disagreement for all items. In addition, benchmark-based evaluation depends on the validity of the dataset answer key. Several responses classified as incorrect by benchmark criteria were considered clinically reasonable by clinician reviewers, suggesting that some apparent errors may reflect limitations of the benchmark rather than deficiencies in model reasoning.

### Future Directions

Future work should expand benchmarking of LRMs with more diverse clinical datasets, multilingual cases, and real-world clinical scenarios to better assess fairness, generalizability, and reasoning robustness [16,34]. Comparative evaluations across multiple models using standardized scenario-level protocols and blinded multi-annotator review will further clarify differences in reasoning behavior and improve methodological reliability [26,29,30]. Exploration of prompt engineering, repeated inference testing, and retrieval-augmented generation approaches may also improve model stability and diagnostic reasoning performance in clinical and educational settings [28,35].

### Conclusions

LRMs demonstrated strong diagnostic performance across complex clinical scenarios in both closed-ended and open-ended question formats. These findings support their potential value as pedagogical and research tools, particularly in supervised settings. Gemini 3 Pro achieved higher overall accuracy than DeepSeek R1. These models show promise as tools for supervised medical education and research. However, further validation is needed using expanded datasets and expert-adjudicated benchmarks. These additional steps, along with validation in real-world clinical environments, will be necessary before such systems can be considered reliable components of clinical decision support.

## Supplementary material

10.2196/76822 Multimedia Appendix 1 Final TRIPOD-LLM mapping table.

10.2196/76822 Multimedia Appendix 2 Complete table of diagnostic accuracy by clinical specialty for DeepSeek R1 and Gemini 3 Pro across closed- and open-ended formats for 6 or more cases per specialty.

## Abbreviations

AI: artificial intelligence
LLM: large language model
LRM: large reasoning model
MCQ: multiple choice question
MedQA: Medical Question Answering
MMLU-Pro: Massive Multitask Language Understanding Pro
TRIPOD-LLM: Transparent Reporting of a Multivariable Model for Individual Prognosis or Diagnosis–Large Language Model
USMLE: United States Medical Licensing Examination
