# Supplementary material for: The Performance of DeepSeek R1 and Gemini 3 in Complex Medical Scenarios: Comparative Study
Source: JMIRx Med. 2026 Apr 27;7:e76822. doi: 10.2196/76822 (PMC13120748; doi:10.2196/76822)
Supplement: Multimedia Appendix 1 [file xmed-v7-e76822-s001.docx]

**Appendix A**

**Final TRIPOD-LLM Mapping Table**

| **Section** | **TRIPOD-LLM Item(s)** | **Description (Short)** | **Where Addressed in Manuscript** |
| --- | --- | --- | --- |
| Title | 1 | Identify the study as LLM evaluation—task, target | Title page, first line |
| Abstract | 2 | Structured abstract (per TRIPOD-LLM) | Abstract section |
| Introduction | 3a | Healthcare context, rationale | Intro, first & second paragraphs |
|  | 3b | Target population/intended use/users | Intro, study aims |
|  | 4 | Objectives & study type | Intro, last paragraph |
| Methods | 5a | Dataset source & rationale | Methods, Data Source |
|  | 5b | Data points/description | Methods, Data Source & Preparation |
|  | 5c | Oldest/Newest item date | Methods, Data Source |
|  | 5d | Pre-processing & quality checks | Methods, Data Source |
|  | 5e | Handling of missing or imbalanced data | Methods, Data Source |
|  | 6a, 6d | LLM name/version, calibration/modifications | Methods, Model Evaluated |
|  | 7c | Annotation/coding protocol | Methods,  Under “Model Evaluated” |
|  | 8, 9a, 9b | Prompt format, instructions, evaluation | Methods, Prompt phases, Protocol |
|  | 12 | Manual execution/no automation | Methods, Evaluation Protocol |
|  | 13 | Ethics, data/code availability | Ethics/Transparency section |
| Results | 7a, 7b | LLM output—accuracy, performance | Results, Model Performance, Table 1 |
|  | 7d | Error breakdown by specialty/type | Results, Error Analysis, Table 2 |
|  | 11, 15, 17 | Statistical results, error codes, qualit. results | Results, Paired Accuracy, Tables 1–3 |
| Discussion | 19a, 19b, 19c, 19g | Interpretation, limitations, challenges, next steps | Discussion, Limitations, Future Directions |
| Declarations | 14a–14f | Funding, conflicts, data/code availability | Transparency/Ethics section |
| References | — | Cited literature | End of manuscript |
