# Supplementary material for: The Performance of DeepSeek R1 and Gemini 3 in Complex Medical Scenarios: Comparative Study
Source: JMIRx Med. 2026 Apr 27;7:e76822. doi: 10.2196/76822 (PMC13120748; doi:10.2196/76822)
Supplement: Multimedia Appendix 2 [file xmed-v7-e76822-s002.docx]

Appendix B

*A Complete Table of Diagnostic Accuracy by Clinical Specialty for DeepSeek R1 and Gemini 3 Pro Across Closed- and Open-Ended Formats for cases 6 and above per speciality*

| Clinical Specialty | DeepSeek R1 (DS) | | Gemini 3 Pro (G) | |
| --- | --- | --- | --- | --- |
|  | Closed-Ended Correct, n (%) | Open-Ended Correct, n (%) | Closed-Ended Correct, n (%) | Open-Ended Correct, n (%) |
| Primary Care (33) | 28 (84.8%) | 26 (78.8%) | 33 (100.0%) | 32 (97.0%) |
| ER Medicine (26) | 25 (96.2%) | 22 (84.6%) | 22 (84.6%) | 23 (88.5%) |
| Pediatrics (17) | 16 (94.1%) | 16 (94.1%) | 15 (88.2%) | 15 (88.2%) |
| OB-GYN (11) | 8 (72.7%) | 7 (63.6%) | 9 (81.8%) | 9 (81.8%) |
| Neurology (10) | 9 (90.0%) | 10 (100.0%) | 10 (100.0%) | 9 (90.0%) |
| Statistics (9) | 7 (77.8%) | 8 (88.9%) | 9 (100.0%) | 7 (77.8%) |
| Infectious Disease (7) | 6 (85.7%) | 4 (57.1%) | 6 (85.7%) | 7 (100.0%) |
| Endocrinology (6) | 6 (100.0%) | 6 (100.0%) | 6 (100.0%) | 6 (100.0%) |
| General Surgery (5) | 2 (40.0%) | 2 (40.0%) | 3 (60.0%) | 4 (80.0%) |
| Gastroenterology (5) | 4 (80.0%) | 3 (60.0%) | 4 (80.0%) | 5 (100.0%) |
| Cardiology (5) | 5 (100.0%) | 4 (80.0%) | 5 (100.0%) | 5 (100.0%) |
| Oncology (5) | 5 (100.0%) | 5 (100.0%) | 5 (100.0%) | 3 (60.0%) |
| Nephrology (3) | 3 (100.0%) | 3 (100.0%) | 3 (100.0%) | 3 (100.0%) |
| Genetics (3) | 1 (33.3%) | 3 (100.0%) | 3 (100.0%) | 3 (100.0%) |
| Psychiatry (4) | 4 (100.0%) | 2 (50.0%) | 2 (50.0%) | 3 (75.0%) |
| Immunology (2) | 2 (100.0%) | 2 (100.0%) | 2 (100.0%) | 2 (100.0%) |
| Allergy Immunology (2) | 1 (50.0%) | 1 (50.0%) | 1 (50.0%) | 2 (100.0%) |
| Trauma Surgery (2) | 2 (100.0%) | 2 (100.0%) | 2 (100.0%) | 1 (50.0%) |
| Rheumatology (2) | 2 (100.0%) | 1 (50.0%) | 2 (100.0%) | 2 (100.0%) |
| Vascular Surgery (1) | 0 (0.0%) | 0 (0.0%) | 1 (100.0%) | 1 (100.0%) |
| Orthopedics (2) | 2 (100.0%) | 2 (100.0%) | 2 (100.0%) | 1 (50.0%) |
| Pulmonology (2) | 2 (100.0%) | 2 (100.0%) | 2 (100.0%) | 1 (50.0%) |
| TOTAL =162 | 140 (86.4%) | 131 (80.9%) | 147 (90.7%) | 144 (88.9%) |
